# Size Aftereffects Are Eliminated When Adaptor Stimuli Are Prevented from Reaching Awareness by Continuous Flash Suppression

**DOI:** 10.3389/fnhum.2017.00479

**Published:** 2017-09-29

**Authors:** Robin Laycock, Joshua A. Sherman, Irene Sperandio, Philippe A. Chouinard

**Affiliations:** ^1^School of Health and Biomedical Sciences, RMIT University, Melbourne, VIC, Australia; ^2^School of Psychology and Public Health, La Trobe University, Melbourne, VIC, Australia; ^3^School of Psychology, University of East Anglia, Norwich, United Kingdom

**Keywords:** adaptation, size aftereffects, continuous flash suppression (CFS), conscious awareness, perception

## Abstract

Size aftereffects are a compelling perceptual phenomenon in which we perceive the size of a stimulus as being different than it actually is following a period of visual stimulation of an adapter stimulus with a different size. Here, we used continuous flash suppression (CFS) to determine if size aftereffects require a high-level appraisal of the adapter stimulus. The strength of size aftereffects was quantified following a 3-s exposure to perceptually visible and invisible adapters. Participants judged the size of a target that followed the adapter in comparison to a subsequent reference. Our experiments demonstrate that the adapter no longer influenced the perceived size of the subsequent target stimulus under CFS. We conclude that the perception of size aftereffects is prevented when CFS is used to suppress the conscious awarness of the adapting stimulus.

## Introduction

Aftereffects are illusory experiences that proceed periods of exposure to certain kinds of sensory input. The prolonged exposure to a sensory input causes neural adaptation, which in turn changes the operating properties of how subsequently presented stimuli are analyzed and perceived (Clifford et al., 2007; Zhao et al., 2011). Neural adaptation leading to aftereffects can occur at many levels of the visual pathway (Webster, 2011). One of the best known aftereffects is the waterfall illusion, which was first described by Addams (1834) after viewing the waterfall of Foyers in Scotland. When Addams turned his gaze away from the waterfall to the vertical face of a nearby cliff, he began to perceive the cliff moving upward in a vivid and realistic manner. Importantly, aftereffects are typically robust and usually occur independently of neuronal fatigue (Virsu, 1978; Thompson and Burr, 2009). Therefore, aftereffects reflect mechanisms directly implicated in fine-tuning the perceptual processing of a sensory stimulus and are not epiphenomenal to a system that has become, in general terms, less responsive to sensory stimulation.

**FIGURE 1:**
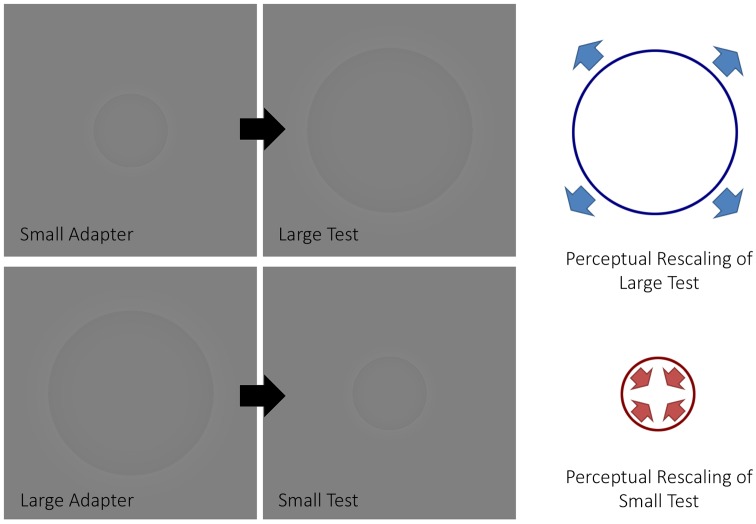
The size aftereffect paradigm. The figure depicts how a size aftereffect paradigm typically works. When a small adapter is presented before a large test stimulus, the latter will appear larger (**top half** of the figure). Conversely, when a large adapter is presented before a small test stimulus, the latter will appear smaller (**bottom half** of the figure).

In a visual psychophysics experiment, Pooresmaeili et al. (2013) demonstrated how size aftereffects can be induced in a manner similar to Addams’ waterfall using Craik-O’Brian-Cornsweet circles (Purves et al., 1999) (**Figure 1**). Namely, an adapter, which had the same diameter across trials, was presented before a test circle. Participants indicated whether the test circle, which varied in size between trials, was smaller or larger than a proceeding reference, which always had a fixed diameter. From these data, points of subjective estimation (PSE) were computed for each participant. The authors observed that the PSE changed depending on whether or not an adapter was presented, such that large adapters decreased the perceived size of the test circles while small adapters increased the perceived size of test circles with respect to when no adapter was presented. That is, the adaptation produced an aftereffect in the opposite direction to that of the adapting stimulus, in a similar fashion that motion aftereffects produce motion in the opposite direction to the adapting motion.

Similar procedures were repeated by Pooresmaeili et al. (2013) in a separate functional magnetic resonance imaging (fMRI) experiment with the exception that participants passively viewed the adapter and test circles. Using a regions-of-interest approach, which only included the analysis of specific visual areas of the brain, the authors demonstrated that activity in the primary visual area (V1) reflected the participants’ perceived rather than retinal size of the test circle. Moreover, the authors demonstrated how a computational model based on local inhibitory mechanisms could account for 81% of variability in their fMRI results. Based on these findings, the authors argued that the observed aftereffects likely originated within V1 and propagated in a feedforward manner. However, current models of visual consciousness emphasize the importance of reverberating loops within the ventral stream (Lamme, 2006) and the amplification of neural activity in visual areas by top–down attention-driven mechanisms (Dehaene and Naccache, 2001). If it is the case that size aftereffects originate within V1 and propagate in a feedforward manner then it is conceivable that processing an adapter by these additional top–down mechanisms, which are thought to be necessary for visual awareness, may not be required for size aftereffects. Thus, the question arises as to whether or not these size aftereffects might survive the suppression of the adapter from conscious awareness.

We performed similar visual psychophysics procedures as those implemented by Pooresmaeili et al. (2013) under continuous flash suppression (CFS) (Tsuchiya and Koch, 2005). CFS is an interocular suppression technique that can be used to examine visual processing outside of awareness. During CFS, the dominant eye views rapidly changing contour-rich patterns, called Mondrians, while the non-dominant eye views a static stimulus of a much weaker contrast. The flickering Mondrians displayed to one eye causes perceptual invisibility of the unchanging stimulus displayed to the other eye. Although the exact mechanisms of this technique are not fully resolved (for reviews, see Sterzer et al., 2014; Yang et al., 2014), there is fMRI evidence for strong suppression of higher-order visual regions during CFS (Cohen et al., 2015; Ludwig and Hesselmann, 2015; Ludwig et al., 2016). The effects of CFS on early visual cortex have been mixed. Some studies suggest that V1 responses are reduced when conscious awareness is suppressed (Yuval-Greenberg and Heeger, 2013; Bahmani et al., 2014) while others suggest that visibility does not significantly modulate V1 responses (Watanabe et al., 2011). Compellingly, multi-voxel pattern analysis (MVPA) is able to decode signals from V1 and successfully discriminate faces from tools (Fogelson et al., 2014), which would not be possible if the visual signal is substantially corrupted at this early level.

While some interocular suppression studies have demonstrated that aftereffects for the orientation of stimuli do not depend on the awareness of the adapter (Wade and Wenderoth, 1978; Bahmani et al., 2014), others have found that aftereffects for more complex features such as face identity, which involve increased processing by higher-level visual areas in the ventral stream, requires awareness of the adapter (Moradi et al., 2005; Amihai et al., 2011). At present, it is unclear whether the perceptual analysis of the size of Cornsweet stimuli might depend on conscious awareness and further processing by higher-level visual areas or can be largely mediated outside of conscious awareness. This study sought to determine if size aftereffects of Cornsweet stimuli can still occur when the adapter is masked by means of CFS. If size aftereffects are mainly mediated by V1 and propagate in a largely feedforward manner as proposed by Pooresmaeili et al. (2013) then its conceivable that they will still be present even when the adpater is outside of conscious awareness.

## Materials and Methods

### Overview

Participants completed one testing session. The testing session began with a handedness questionnaire followed by tests that assessed visual acuity, eye dominance, and ability to fuse stimuli binocularly. This was followed by a threshold experiment to determine the Michelson luminance contrast required for an adapter to break CFS. The participant then completed the aftereffects experiment with the luminance contrast for the adapter stimuli adjusted so that it was below the threshold value obtained in the former experiment. The aftereffects experiment used a paradigm similar to the one described in Pooresmaeili et al. (2013) but also included additional conditions with CFS. We used the method of constant stimuli (Ehrenstein and Ehrenstein, 1999) to obtain psychometric curves and calculate points of subjective equality (PSE) and bistability widths (ω) from these curves. The former consists of a measure of perceived size while the latter consists of a measure of perceptual uncertainty (Wood et al., 2016). The testing session ended with a second threshold experiment to verify that the luminance contrast used for adapter stimuli under CFS in the aftereffects experiment remained below threshold. In total, the testing session took 2 h to complete with breaks. This study was carried out in accordance with the recommendations of the La Trobe University Human Ethics Committee with written informed consent from all participants. All participants gave written informed consent in accordance with the Declaration of Helsinki. The protocol was approved by the La Trobe University Human Ethics Committee.

### Participants

Twenty-four adults participated in the study. We randomly assigned participants to one of two conditions: the *small* or *large adapter* conditions. Twelve participants completed the *small adapter* condition (6 males, age range 18 – 51, *M* = 25.5 years, *SD* = 9.1) and 12 participants completed the *large adapter* condition (6 males, age range 20 – 33, *M* = 25.7 years, *SD* = 3.9). To be included in the study, participants needed to be right handed and have normal or corrected-to-normal vision. We verified handedness using a modified version of the Edinburgh Handedness Inventory Questionnaire (Oldfield, 1971). All participants received a score of 90% or higher. We screened for binocular dysfunction by asking participants to place their index finger at arm’s length between their eyes and a single target on the wall two meters away. Participants were then instructed to focus their gaze on their finger and report how many targets on the wall they perceived. Then, participants were instructed to focus their gaze on the distant target and report how many fingers they perceived. Binocular fusion was deemed intact if the participant said *two* in both cases. Also, the Snellen chart was used to confirm that each eye had at least 20/20 vision. In addition, eye-dominance was assessed using the Miles test (Miles, 1930). This test was conducted by asking participants to make a diamond with their hands by joining both their index fingers and thumbs together. With arms outstretched, participants viewed a distant target through this aperture and gradually brought their hands toward their face. The eye to which participants brought their hands toward while still being able to view the distant shape was determined as the dominant eye. The Miles test revealed left-eye dominance in 5 participants and right-eye dominance in 19 participants. None of the participants had trouble with binocular fusion.

### Stimuli and Apparatus

The stimuli consisted of Craik-O’Brian-Cornsweet circles (Purves et al., 1999). We created the stimuli in MATLAB (Math Works, Natick, MA, United States) using procedures similar to those described elsewhere (Purves et al., 1999). As shown in **Figure 1**, the center of the stimuli appeared darker relative to the background – although both were physically isoluminant (luminance: 40.3 c/m2, RGB: 128, 128, 128). Stimuli were presented using E-Prime 2 software (Psychology Software Tools, Sharpsburg, PA, United States) on a 23″ liquid crystal display (LCD) computer screen with a resolution of 1600 × 900 pixels. The frame rate of the monitor was 60 Hz. Participants viewed the stimuli through a mirror stereoscope with their heads on a chin rest. Viewing distance from the eyes to the computer screen was 57 cm. To aid binocular fusion, stimuli displayed to both eyes were each centered inside a pictorial frame subtending 17° by 17° of visual angle behind a fixation point.

### Procedures for the Threshold Experiment

For each trial, we presented a stimulus with a Michelson luminance contrast of 2, 3, 4, 5, 6, 7, or 8% under CFS for 3,000 ms (for details regarding CFS, see Procedures for the Size Aftereffects Experiment). The stimulus subtended either 4° or 9° of visual angle depending on whether the participant was assigned to the *small* or *large adapter* conditions. Participants indicated verbally whether or not they had *any* inclination of seeing a stimulus being presented under CFS. Each luminance contrast condition was tested five times. We also included ten catch trials without a stimulus under CFS. E-Prime randomly generated the presentation order of the different conditions. We defined the participant’s perceptual threshold as the lowest luminance contrast for which a stimulus broke suppression on a single trial or more. A threshold of 8%, the highest contrast value tested, was assigned to those participants who did not experience any breakthrough in suppression during the threshold experiment.

### Procedures for the Size Aftereffects Experiment

Participants in both the *small* and *large adapter* conditions completed four blocks. These blocks consisted of: (1) a visible adapter stimulus preceding a target, (2) no adapter preceding a target, (3) an adapter stimulus under CFS preceding a target, and 4) no adapter under CFS preceding a target. We counterbalanced the order in which participants completed the blocks. E-prime randomly generated the order of trials within each block. We set the luminance contrast for all stimuli (i.e., the adapter, target, and reference stimuli) 1% below the participant’s threshold (i.e., a 3% threshold meant that all experimental stimuli were adjusted to a 2% luminance contrast). The exception to this rule was that all participants with a threshold larger than 5% luminance contrast had the stimuli adjusted to 4% luminance contrast. This approach helped maximize the effects of the adapter stimulus while ensuring that the stimulus remained outside of conscious awareness (Stein and Sterzer, 2011).

**FIGURE 2:**
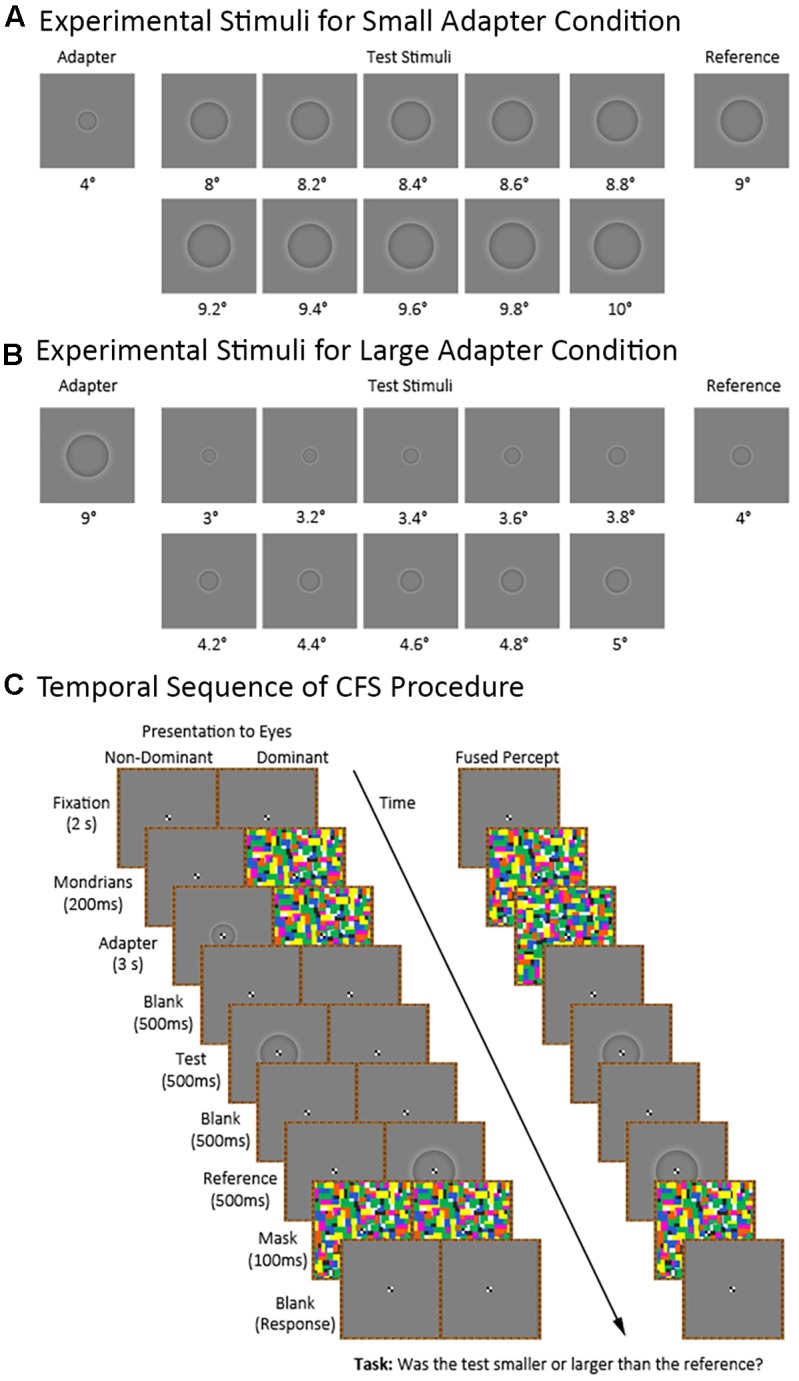
Continuous flash suppression (CFS) procedure and experimental stimuli for the small and large adapter conditions. The figure shows the stimuli used in the *small* **(A)** and *large* **(B)** adapter conditions. Also shown **(C)** is the sequence of stimuli in the *adapter present* condition presented to the non-dominant and dominant eyes as well as the participant’s expected fused perceptual experience.

**Figure 2** shows the temporal sequence of events for a trial in the *CFS* condition (the *visible* condition was identical to the *CFS* condition except for the presentation of the flashing Mondrians). Each trial began with the participant maintaining central fixation over a blank image for 2,000 ms. Afterwards, we presented a series of Mondrian images to the dominant eye at a frequency of 10 Hz (Tsuchiya and Koch, 2005) for 3,200 ms. The series of Mondrian images consisted of eight pictures cycling in a sequential order for a total of 32 presentations. We presented the Mondrian images to the dominant eye because suppression of conscious awareness works better for information presented to the non-dominant compared to the dominant eye (e.g., Almeida et al., 2008; Laycock et al., 2017). Meanwhile, the non-dominant eye was presented with either a blank image for 3,200 ms in the *adapter absent* condition or a blank image for 200 ms followed by an adapter stimulus for 3,000 ms in the *adapter present* condition. The latter was to ensure that the Mondrians presented to one eye had already directed the participant’s attention before presenting the adapter stimulus to the other eye. The adapter stimulus subtended 4° and 9° of visual angle during the *small* and *large adapter* conditions, respectively.

Afterwards, we presented a blank image to both eyes for 500 ms followed by a test stimulus to the non-dominant eye for 500 ms. The test stimulus ranged in size between trials. For the *small adapter* condition, the test stimulus subtended between 8° to 10° of visual angle with 10 different possible sizes varying in 0.2° increments. For the *large adapter* condition, the test stimulus subtended between 3° to 5° of visual angle with ten different possible sizes varying in 0.2° increments. E-prime randomly generated the order of presentation for the different test sizes.

Finally, we presented a blank image for 500 ms followed by a reference stimulus for 500 ms to the dominant eye. The reference stimulus subtended 9° and 4° of visual angle in the *small* and *large adapter* conditions, respectively. An interstimulus interval (ISI) of 500 ms between the adapter and test stimuli was chosen in light of an earlier study demonstrating strong aftereffects at this ISI (Suzuki and Cavanagh, 1998). We then presented a mask to both eyes for 100 ms. After the mask disappeared, participants verbally indicated whether they perceived the test stimulus as “smaller” or “larger” than the reference stimulus. The experimenter manually entered the participant’s response using a keyboard.

### Data Analyses

We carried out statistical analyses using the Statistical Package for the Social Sciences version 23 (SPSS; IBM Corporation; Armonk, NY, United States), JASP software version 0.8 (University of Amsterdam, Amsterdam, Netherlands), and GraphPad Prism version 6 (GraphPad Software, Inc.; La Jolla, CA, United States). For the threshold experiment, we performed an analysis of variance (ANOVA) with Time (*pre* vs. *post*) as a within-subject factor and Adapter Size (*small* vs. *large*) as a between-subject factor. We further verified that each participant’s threshold in the *post* condition remained above the luminance contrast selected for the size aftereffects experiment.

For the size aftereffects experiment, we created psychometric curves for each condition in each participant based on their responses. This was done by counting the number of times the participant reported the target stimulus as appearing “larger” than the reference stimulus. Using the following logistic function, we calculated the probability (*P)* of the participant reporting the test stimulus as appearing larger than the reference:

$$P(x)=\left(\frac{e^{b_{0}+b_{01}x}}{1+e^{b_{0}+b_{1}x}}\right)$$

Where *b*_0_ and *b*_1_ are coefficient estimates based on an initial general linear model (binary logit) fit. From this function, the PSE was calculated as *P* = 0.5, representing how large the target stimulus needed to be for the participant to judge this stimulus as having the same apparent size as the reference stimulus with *higher PSE values signifying that the test stimulus was perceived as smaller*. Additionally, we calculated the bistability width (ω) as:

$$\omega =P_{0.75}-P_{0.25}$$

Where *P*_0.25_ and *P*_0.75_ correspond to *P* = 0.25 and *P* = 0.75, respectively. In this case, the bistability width (ω) provides a measure of variability in the perceived size judgments by the participant with *higher ω values signifying greater perceptual uncertainty*. After extracting the PSE and ω values, we performed ANOVA on these dependent variables with Adapter Presence (*adapter present* vs. *adapter absent*) and Perceptual Visibility (*No CFS* vs. *CFS*) as within-subject factors and Adapter Size (*small adapter* vs. *large adapter*) as a between-subject factor. Simple effect tests and Bonferroni-corrected pairwise comparisons were used to further analyze significant interactions. One sample *t*-tests against the physical size of the reference were further used to confirm the presence of aftereffects. Unless specified otherwise, all reported *p*-values are based on two-tailed criteria and corrected for multiple comparisons. For effect sizes, we report the partial eta squared ($\eta_{p}^{2}$) values obtained from the ANOVA and calculated Cohen’s *d* for pair-wise comparisons as the difference between the two means divided by their pooled standard deviation (Cohen, 1988).

In addition to null hypothesis statistical testing, which does not allow one to draw definite conclusions about the viability of the null hypothesis, we calculated Bayes Factors (*BF*_10_) denoting the likelihood of the alternative (*H*_1_) over the null (*H*_0_) hypothesis. *BF*_10_ indicates more evidence in support of the alternative hypothesis as it increases and more evidence in support of null hypothesis as it decreases. We considered *BF*_10_ values of 3 or more to provide substantial support for the alternative hypothesis and *BF*_10_ values less than 0.33 to provide substantial support for the null hypothesis (Wetzels et al., 2011).

## Results

### Threshold Experiment

**FIGURE 3:**
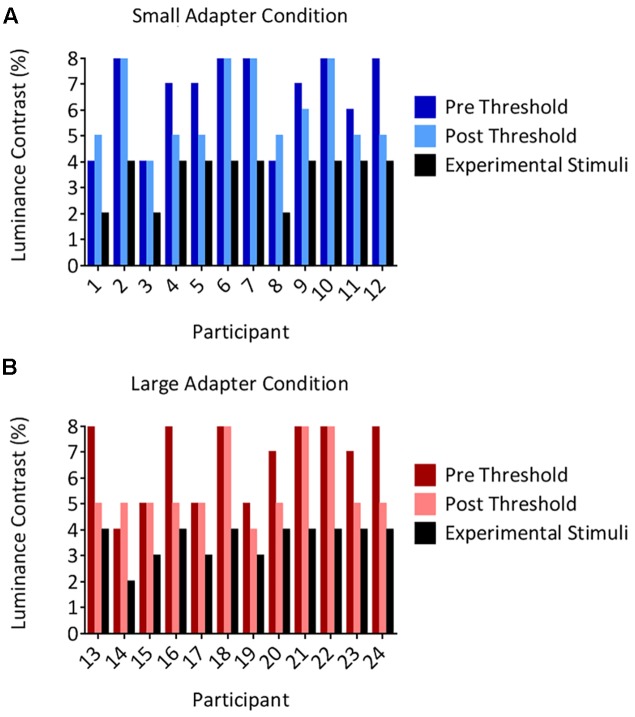
Individual suppression thresholds. The figure shows thresholds measured before (the *pre* condition in dark color columns) and after (the *post* condition in light color columns) the size aftereffects experiment, for both the *small* **(A)** and *large* **(B)** *adapter* conditions. The *x*-axes represent the participant number and the *y*-axes represent the luminance contrast (%). Thresholds were defined as the lowest luminance contrast for which a stimulus broke suppression on any trial. Black columns denote the luminance contrast used to present the adapter in the size aftereffects experiment. Importantly, these values remained below the *post* threshold measurements in every participant. Thus, participants should not have ever been aware of the presence of the adapter stimulus in the size aftereffects experiment. This was subsequently confirmed by every participant reporting not having seen an adapter break suppression in the *CFS* condition in the size aftereffects experiment.

The threshold luminance required to maintain suppression decreased following the aftereffects experiment. Our analyses demonstrated a main effect of Time [*F_(1,22)_* = 9.05, *p* = 0.006, $\eta_{p}^{2}$ = 0.29] but not a main effect of Adapter Size [*F_(1,22)_* < 0.01, *p* = 0.956, $\eta_{p}^{2}$ < 0.01] nor an interaction between Time and Adapter Size [*F_(1,22)_* = 1.38, *p* = 0.252, $\eta_{p}^{2}$ = 0.06]. Although the decreases in thresholds were statistically different, they were not meaningfully different, reducing on average by 0.96% in luminance contrast. Inspection of the individual data revealed that none of the participants in either the *small* (**Figure 3A**) or *large* (**Figure 3B**) *adapter* conditions had a *post* threshold that was less than or equivalent to the luminance contrast that we used to present adapters in *CFS* condition during the aftereffects experiment. In addition, no participant ever reported seeing an adapter breaking suppression during the aftereffects experiment. Taken together, this evidence demonstrates that the adapters remained perceptually invisible in the *CFS* condition even though the thresholds decreased slightly after the aftereffects experiment.

### Size Aftereffects Experiment: Goodness of Fit in the Psychometric Curves

We assessed the goodness of fit in the resulting psychometric curves in each participant for each of the different conditions. Participants in the *small* (mean *R^2^* = 0.91, *SD* ± 0.04, range = 0.79–0.99) and *large* (mean *R^2^* = 0.89, *SD* ± 0.05, range = 0.75–0.98) adapter showed good fits across all conditions.

### Size Aftereffects Experiment: Analysis of PSE Values

**FIGURE 4:**
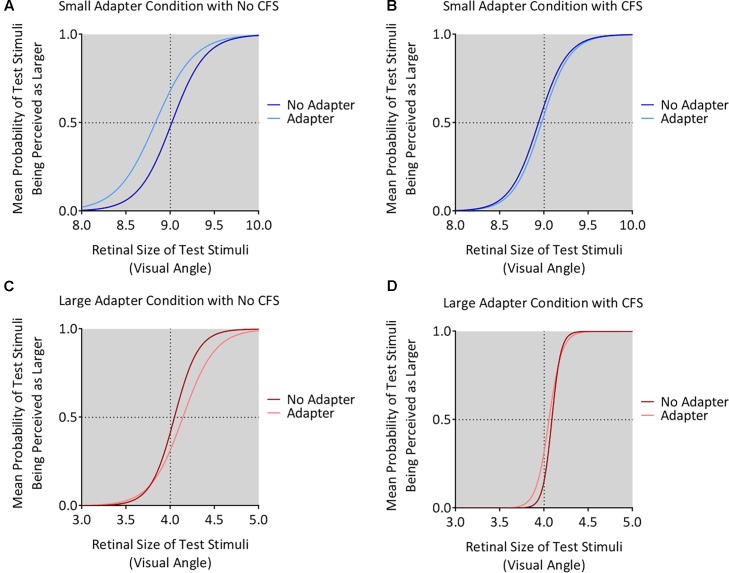
Mean psychometric function curves for all conditions. The figure shows the mean psychometric curves for participants in the *small adapter* condition during the *No CFS* **(A)** and *CFS* **(B)** conditions. The figure also shows the mean psychometric curves for participants in the *large adapter* condition during the *No CFS* **(C)** and *CFS* **(D)** conditions. Dark curves correspond to the *adapter absent* condition while light curves correspond to the *adapter present* condition. The *x*-axes correspond to the test stimulus size in visual angle (1° corresponding to 1 cm in physical size) while the *y*-axes correspond to the probability (*P*) that the test stimulus appeared larger than the reference stimulus. The dashed horizontal lines denote the actual size of the reference stimulus while the dashed vertical lines denote the points of subjective estimation (PSE) defined as *P* = 0.5. The PSE represents how large the target stimulus needed to be for the participant to judge this stimulus as having the same apparent size as the reference stimulus. Conceptually, this means higher PSE values for when the test stimulus was perceived smaller.

**FIGURE 5:**
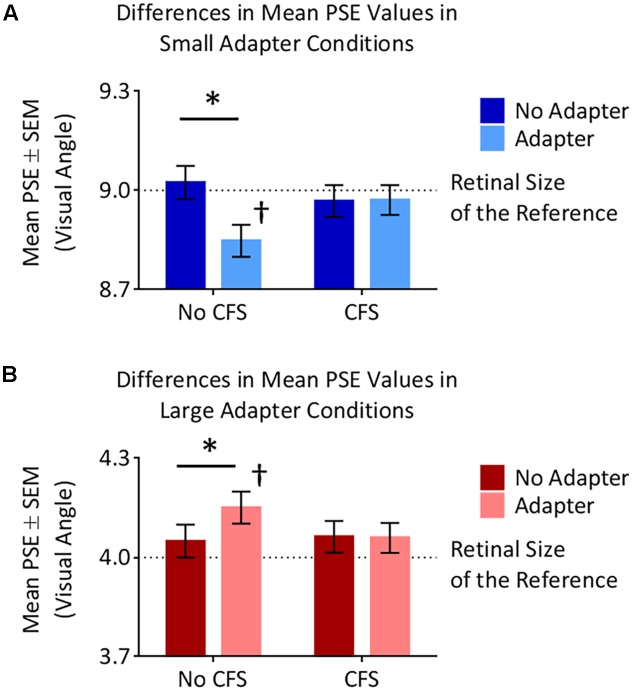
Points of subjective estimation (PSE) for the different conditions. **(A)** In the *small adapter* condition, participants perceived the test stimulus as larger, as reflected by smaller PSE values, when the adapter was present versus absent in the *No CFS* (^∗^) but not the *CFS* condition. PSE values in the *No CFS* condition were also lower than the retinal size of the reference stimulus (†). **(B)** In the *large adapter* condition, participants perceived the test stimulus as smaller, as reflected by larger PSE values, when the adapter was present versus absent in the *No CFS* (*^∗^*) but not the *CFS* condition. PSE values in the *No CFS* condition were also higher than the retinal size of the reference stimulus (†).

The small adapter made the test stimulus appear larger while the large adapter made the test stimulus appear smaller, but only when these adapters were visible to the participant (**Figures 4**, **5**). ANOVA demonstrated a three-way interaction between Adapter Presence, Perceptual Visibility, and Adapter Size [*F_(1,22)_* = 8.51, *p* = 0.008, $\eta_{p}^{2}$ = 0.28]. Deconstructing this interaction revealed differential effects of Adapter Size when the adapter was present in the *No CFS* but not the *CFS* conditions (**Figure 5**).

Comparing the *small adaptor present* with the *small adaptor absent* conditions demonstrated that participants perceived the test stimulus as larger (demonstrating smaller PSE values) in the *No CFS* condition (*p* = 0.001, *d* = 0.80, *BF*_10_ = 9.75, see asterisks in **Figure 5A**) when the adapter was consciously visible, but not in the *CFS* condition (*p* = 0.940, *d* = 0.02, *BF*_10_ = 0.29) when the adapter was presented outside of conscious awareness. One-sample t-tests confirmed that PSE values in the *No CFS* (*p_uncorr_* = 0.032, *d* = 0.71, *BF*_10_ = 2.32, see dagger in **Figure 5A**) but not in the CFS (*p_uncorr_* = 0.599, *d* = 0.16, *BF*_10_ = 0.33) conditions were significantly lower than the retinal size of the reference stimulus.

Conversely, in the *large adapter present* condition, participants perceived the test stimulus as smaller (demonstrating larger PSE values) compared with the adaptor *absent* condition in the *No CFS* (*p* = 0.041, *d* = 1.01, *BF*_10_ = 2.28, see asterisks in **Figure 5B**) but not the *CFS* (*p* = 0.937, *d* = 0.03, *BF*_10_ = 0.29) conditions. One-sample *t*-tests confirmed that PSE values in the *No CFS* (*p_uncorr_* < 0.001, *d* = 1.51, *BF*_10_ = 120.56, see dagger in **Figure 5B**) but not *CFS* (*p_uncorr_* = 0.116, *d* = 0.49, *BF*_10_ = 0.89) conditions were significantly higher than the retinal size of the reference stimulus.

### Size Aftereffects Experiment: Analysis of ω Values

**FIGURE 6:**
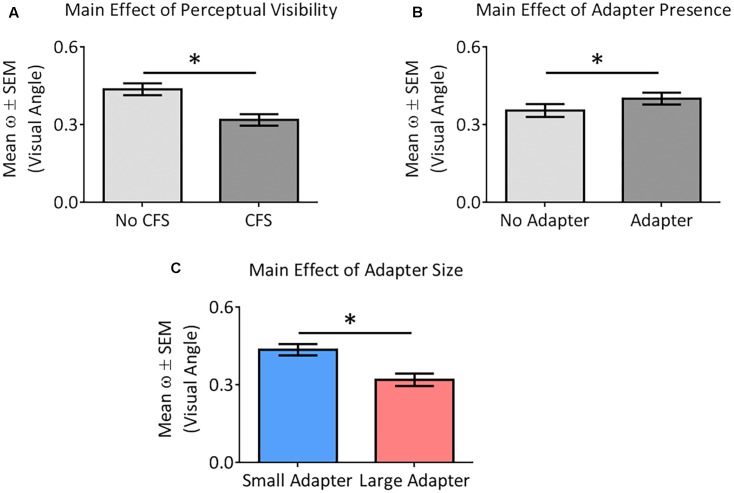
Main effects of the different factors on perceptual certainty (ω). Our analysis demonstrated perceptual uncertainty in the *No CFS* relative to the *CFS* condition **(A)**, greater perceptual uncertainty in the *adapter present* relative to the *adapter absent* condition **(B)**, and greater perceptual uncertainty in the *small adapter* relative to the *large adapter* condition **(C)**. Asterisks (^∗^) denote significant differences at *p* < 0.05.

ANOVA demonstrated greater perceptual uncertainty in the *No CFS* relative to the *CFS* condition [*F_(1,22)_* = 19.37, *p* < 0.001, $\eta_{p}^{2}$ = 0.47], greater perceptual uncertainty in the *adapter present* relative to the *adapter absent* condition [*F_(1,22)_* = 4.85, *p* = 0.038, $\eta_{p}^{2}$ = 0.18], and greater perceptual uncertainty in the *small* relative to the *large adapter* condition [*F_(1,22)_* = 6.83, *p* = 0.016, $\eta_{p}^{2}$ = 0.24] (**Figure 6**). ANOVA did not reveal any interaction between these factors (all *p* > 0.09, all $\eta_{p}^{2}$ < 0.13).

## Discussion

We tested whether or not size aftereffects requires conscious awareness of the adapting stimulus. If such effects are independent of conscious awareness then we would expect to see aftereffects regardless of the visibility of the adapting stimulus. Instead, the results demonstrated that the suppression of an adapting stimulus from conscious awareness by means of CFS completely eliminated size aftereffects. That is, size aftereffects were established when participants were consciously aware of the adapter but not when they were consciously unaware of the adapter. Given that aftereffects without awareness of the adapting stimulus are generally reported for stimuli associated with early but not with later visual processing (Yang et al., 2014) – a view consistent with models suggesting that interocular suppression deepens as the signal progresses further along the visual hierarchy (Nguyen et al., 2003) – we conclude that the perceptual analysis of the size of Cornsweet stimuli may require a level of processing beyond the earlier stages of visual cortical processing.

### Interpreting Perceived Size and Perceptual Certainty Results

We used the PSE dependent variable to assess the perceived size of the target stimulus. The results reveal that participants perceived the target stimulus differently only when an adapter was presented before it and when this adapter was consciously seen. When this adapter was not consciously seen as a result of CFS, it did not affect the perceived size of the target stimulus. This null finding is unlikely the result of an insufficient number of participants when one considers both the reported effects sizes, which tend not to vary in a meaningful way with the inclusion of more participants, and the Bayesian analyses carried out to compliment the more traditional null statistical hypothesis testing performed. Specifically, the presence versus absence of the adapter had negligible effect sizes in the *CFS* condition (*d* ≤ 0.04) and large effect sizes in the *No CFS* condition (*d* ≥ 0.80) while the reported Bayesian factors for the former indicated substantial support for the null hypothesis (*BF*_10_ = 0.29) (Wetzels et al., 2011). Thus, we conclude that an adapter requires conscious awareness to exert an effect on the size of a subsequent target stimulus. In other words, for size aftereffects to occur, the adapter needs to be processed within consciousness, though importantly, this conclusion only applies when conscious awareness is manipulated by interocular suppression. Further testing will be required to determine if this also holds for other methods of suppressing conscious awareness, such as backward masking for example.

In contrast, we measured the *ω* dependent variable to assess perceptual certainty. This measurement increases as a function of cognitive demands and neural activation related to the processing of information (Wood et al., 2016). In this study, we demonstrate higher *ω* values in the *adapter present* relative to the *adapter absent* condition, which most likely reflects additional processing related to a second stimulus in the former condition as opposed to only one stimulus in the latter condition. This same explanation may also help clarify why we demonstrated higher *ω* values in the *CFS* compared to the *No CFS* condition. Namely, during CFS, participants were not consciously aware of an adaptor stimulus and thus only consciously saw the target stimulus prior to the reference. On the other hand, without CFS, participants viewed the adaptor followed by the test and finally the reference which may have required additional cognitive processes and resulted in more perceptual uncertainty. Last, we demonstrate higher *ω* values in the *small* versus *large adapter* condition. We suggest that this could relate to a more thorough perceptual analysis of the small adapter as a result of it being confined to the fovea. The edges of the large adapter, in contrast, were located outside of central vision. fMRI studies reveal that more neural resources are devoted to processing the edges of smaller than larger rings due to cortical magnification (e.g., Fang et al., 2008).

### Could a Model Involving Only V1 Mechanisms Explain Size Aftereffects?

Pooresmaeili et al. (2013) tested several models to account for their fMRI data and proposed that a bottom-up explanation best explained the variability mediating their observed size aftereffects. However, they did not consider that fMRI has a number of limitations that make distinctions between bottom-up versus top–down mechanisms difficult to establish with certainty. First, fMRI lacks the temporal resolution (∼4-6 s) to rule out the possibility that higher-order visual areas may still have modulated the observed activity within V1 via feedback mechanisms, even when the fMRI data is entered into various models that try to dissociate between bottom-up versus top–down modulation. Alternative brain imaging techniques with superior temporal resolution are ultimately required to confirm this distinction in processing. Second, while V1 is necessary for conscious sight (e.g., Leopold, 2012), this does not imply that V1 is sufficient for perception. Brain stimulation studies that are able to make more causal statements have determined that disruption of occipito-temporal cortex can reduce the Müller-Lyer illusion (Mancini et al., 2011) and that feedback projections from higher-order visual regions into V1 are required for conscious perception (Pascual-Leone and Walsh, 2001). Of direct relevance to the current results, transcranial direct current stimulation of the early visual cortex disrupted size judgment perception due to an interference of top-down modulation from higher-order areas (Costa et al., 2015).

### Early Visual Areas Still Process Retinal Information Outside of Conscious Awareness

One should consider to what extent the adapting stimulus was processed by the brain during CFS. Our experimental paradigm allowed for plenty of opportunity for early visual pathways to process the adapter and consequently influence the perceived size of the test stimulus prior to entry into the cortex. This is because both the adapter and test stimuli were presented to the same eye. The seminal work of Hubel and Wiesel (1962) demonstrates that information from each eye remains fully segregated in the early visual pathways before entering V1, with neurons in the lateral geniculate nucleus having monocular responses. While convergence from both eyes begin in V1, some monocular selective responses are still observed in this region (Tootell et al., 1988). Thus, considering this anatomical organization of the early visual pathways, there is little reason to believe that retinal information from the suppressed eye would not reach V1 unless feedback mechanisms were at play to gate information from the suppressed eye from reaching the cortex. However, this seems unlikely. A number of fMRI studies have revealed that retinal information suppressed from conscious awareness is still processed by V1 under conditions of CFS while the processing of this same information is absent in other higher-order visual regions (Hesselmann and Malach, 2011; Troiani and Schultz, 2013; Yuval-Greenberg and Heeger, 2013; Fogelson et al., 2014).

For example, as mentioned earlier in the Introduction, MVPA of fMRI signals has demonstrated how retinal information from the suppressed eye is processed by early but not late visual cortical areas (Fogelson et al., 2014). In this study, the authors presented faces and tools under CFS and no CFS conditions. MVPA was used to decode the fMRI signal to determine the category of the stimuli presented to the participants, namely a face or a tool. Without CFS, MVPA could successfully decode the signal in a network of areas from occipital, temporal, parietal and frontal cortices. However, only the early visual areas in the occipital cortex could reliably classify the stimuli under CFS conditions. Given that the higher order areas, such as medial occipital, lateral temporal, and lateral frontal cortex, were unable to decode the invisible stimuli, the results suggested an association between awareness and neural processing in the ventral temporal cortex and other areas in the brain. Although some other studies have demonstrated higher-order ventral stream activation for invisible stimuli during CFS (e.g., Sterzer et al., 2009), Noguchi et al. (2012) showed that activation in more ventral visual areas was only evident within the first 200 ms, perhaps suggesting that sustained processing within the ventral stream is required for conscious awareness.

In addition, single-cell electrophysiology studies in the monkey have shown that almost all monocular cells, and the vast majority of V1 cells tested, were not affected by suppression from awareness (Leopold and Logothetis, 1996). For example, it has been shown that CFS does not alter spiking activity in V1 to adapter or prime stimuli (Wilke et al., 2006) but it does alter spiking activity in higher-level visual areas in the ventral stream (Sweeny et al., 2011). Interestingly, studies on binocular rivalry yield opposing findings depending on the brain imaging technique used. For example, electrophysiological measurements of spiking high frequency local field potentials were unaffected by suppression from awareness while BOLD activation in V1 was reduced in the same monkey participants (Maier et al., 2008). The authors of this study suggested that this could reflect recurrent processing from higher-order visual regions into V1 driven by attentional modulation and awareness (Maier et al., 2008). This view of the importance of feedback signals to V1 for conscious awareness has also been argued elsewhere (Tong, 2003) and may explain why some fMRI studies show reduced V1 activation under CFS conditions (e.g., Troiani and Schultz, 2013; Yuval-Greenberg and Heeger, 2013). Reduced V1 responses may not indicate the absence of processing of retinal information but may rather reflect the absence of signal amplification from recurrent processing to the same degree as would be expected if processed within conscious awareness.

### Does the Cornsweet Illusion Require Conscious Processing?

One should also consider whether or not a complex analysis required to perceive the Cornsweet stimuli is achievable under conditions of CFS. The illusion of luminance contrast created by these stimuli is typically regarded as being mediated by early visual processes occurring at the level of V1 or even LGN (Anderson et al., 2009). It is possible that although retinal inputs from the suppressed eye reached at least as far as V1, the current results may reflect the inability of the Cornsweet illusion to be detected without awareness rather than an absence of size aftereffects *per se*. Indeed, Masuda et al. (2011) presented the edge of a Cornsweet illusion to participants under backward masking and CFS conditions and revealed that one must have a conscious awareness of the edge to experience a real-time illusion of differing luminance either side of the edge. More critically, in a separate adaptation experiment, the authors briefly applied CFS to the edge of the illusion and then had participants view a gray background. In this condition, the participants saw the illusion proceeding the edge presented under CFS. Thus, although the participants were not consciously aware of the Cornsweet stimulus, the fact that the illusion occurred after CFS without the awareness of the Cornsweet edge indicates that the local gain mechanism in V1 did process the border. These results indicate that although the perception of the Cornsweet illusion disappears without awareness of the edge, suggesting the necessity of higher-order processing, the edge is nonetheless still processed at monocular stages of processing. Masuda et al. (2011) proposed two mechanisms to be involved in the Cornsweet illusion: a feedback mechanism associated with the subjective awareness of the edge followed by a mechanism for surface lightness, which may rely on lateral connectivity being activated by the feedback signal. For the current purposes, the results from Masuda et al. (2011) indicate that on the one hand early visual regions can process Cornsweet circles even when suppressed from awareness. On the other hand, the experiments reported by Masuda et al. (2011) reinforce the suggestion that top–down signals associated with conscious awareness appear to be necessary for higher-order processing of Cornsweet circles as required for aftereffects.

To process and adapt to the size of Cornsweet stimuli, it is conceivable that the entire form must be processed as a Gestalt, and thus we consider the contribution of global processing to the current results. It may be that CFS prevented such global perceptual processing to occur. Global, as against more detailed or local processing, is a function of higher-order visual areas with larger receptive fields than in V1 (Van Essen and Gallant, 1994; Badcock and Clifford, 2004). However, the CFS literature on global visual processing has often highlighted the presence of global processing when awareness is suppressed. For example, in a CFS study, global motion and form coherence patterns broke suppression faster with higher coherence, which may indicate processing of global information without awareness (Chung and Khuu, 2014). Although perceiving the direction of illusory triangles (i.e., Kanizsa stimuli formed by Pacmen-like inducers) has been shown to be at chance levels when the inducing stimuli are rendered invisible by CFS (Harris et al., 2011), the time for the Kanizsa triangles to break suppression and reach conscious awareness was faster than when the inducer stimuli were rotated randomly (Wang et al., 2012). It is important to note that the extent to which breaking CFS studies shed light on non-conscious processes has been challenged (Stein and Sterzer, 2014) – though the findings from Wang et al. (2012) appear to accord with recommendations made by Gayet et al. (2014) for the sound design of breaking CFS experiments. The findings of Wang et al. (2012) may suggest that some forms of perceptual grouping occur without conscious awareness. However, conflicting results of the ability to process facial expression without awareness has been reported. While Faivre et al. (2012) demonstrated that emotional faces suppressed by CFS were unable to bias preference judgments of unfamiliar Chinese character (i.e., pleasant, unpleasant judgments), Almeida et al. (2013) were able to demonstrate that angry faces suppressed by CFS made similar ratings less favorable.

Thus, although there is some mixed evidence, it appears that a wide array of global or Gestalt processing can be achieved without conscious awareness. One potential explanation for the apparent lack of global processing in the current study may be the type of stimulus used. Perhaps the Cornsweet illusion comprises a more basic stimulus that does not require the same type of integration of local features as would be required for motion and form coherence, faces, and illusory shape perception.

### The Importance of the Ventral Stream for Conscious Sight

There are multiple lines of evidence that the ventral stream is important for conscious sight. For example, Jiang and He (2006) have shown how faces under CFS conditions were no longer processed in the fusiform face area (FFA) despite these images still being processed in the subconscious as evidenced by significant activations in both amygdala and the superior temporal sulcus area (STS). In a different fMRI study, Cohen et al. (2015) examined how activation in different regions of interest correlated with the breaking of suppression of different categories of objects. They found a tighter coupling between activation and abilities in seeing categories of objects breaking suppression in ventral stream areas, in particular the lateral and ventral occipital-temporal areas, than they did in the early visual cortex.

Thus, the emerging view from studies of conscious and subconscious processing, including those utilizing CFS to suppress visual awareness, is one that highlights an important role of sustained ventral stream processing, and activation throughout the entire brain in conscious awareness as opposed to the idea that certain percepts, such as size (Pooresmaeili et al., 2013), are largely mediated by local mechanisms confined to V1. The importance of multiple brain areas in mediating perception is in agreement with multiple theories of visual consciousness. For example, Lamme’s theory stipulates that visual mechanisms work to process information in a reverberating manner to assign meaning to the representation (Lamme and Roelfsema, 2000; Lamme, 2006). This information needs to be integrated across multiple areas, and specialized regions are tasked to assign a particular meaning for a more complex analysis. A different theory, global work space theory, proposes that the conscious awareness of an object corresponds to the long range broad casting of information across the brain with preconscious visual information able to enter this workspace (Dehaene et al., 2003).

### Manipulating Conscious Awareness of Adapting Stimuli in Other Aftereffect Paradigms

Psychophysics paradigms have explored over a number of decades the contribution of conscious awareness to other types of visual aftereffects, utilizing interocular techniques such as CFS and binocular rivalry. Many studies have demonstrated that early visual mechanisms are amenable to adaptation aftereffect even without perceptual awareness. For example, the magnitude of the tilt (Wade and Wenderoth, 1978) and motion aftereffects (Lehmkuhle and Fox, 1975; O’Shea and Crassini, 1981) are unaffected by the duration of suppression of the adapting stimulus, using binocular rivalry. These studies argued that the site of suppression must be after the site of the aftereffect.

Maruya et al. (2008) used static and dynamic test stimuli under CFS to suppress motion adaptors allowing exploration of motion aftereffects presumed to reflect, respectively, low-level and high-level cortical visual processes. With adaptor and test stimuli presented to the same eye, both static and dynamic motion aftereffects were found, although attenuated. With adapter and test presented to opposite eyes, there were no static motion aftereffects for visible or invisible conditions, suggesting that interocular transfer requires higher-level processing. The dynamic motion aftereffect was completely reduced in the invisible compared to the visible condition, with the authors concluding there is no adaptation of higher-level systems without conscious awareness of the motion adaptor.

Similarly, face processing, including face identity (Moradi et al., 2005, using binocular rivalry) and subordinate information about a face, such as gender or race (Amihai et al., 2011, using CFS), require higher order visual processing and only show aftereffects with conscious awareness of the adapting stimulus. In other words, it may be that aftereffects without conscious awareness of adapting stimuli are only possible with low-level visual processes, as suggested by the evidence for face shape aftereffects surviving interocular suppression (Stein and Sterzer, 2011). Hence, in the context of the size aftereffects measured in the current study, it appears that higher-order ventral stream adaptation to Cornsweet illusion size information (Grill-Spector et al., 1999; Eger et al., 2008) could not take place once conscious awareness was suppressed by CFS.

In contrast, a consistent correspondence between higher- and lower-level visual processing and the existence of aftereffects without conscious awareness are not apparent. For example, both linear and spiral motion aftereffects have been demonstrated when adapters were suppressed from awareness by CFS (Kaunitz et al., 2011), with only spiral motion assumed to require higher-level dorsal stream (MT/MST) visual processing. Intriguingly, Adams et al. (2010) have used CFS to demonstrate the presence of face emotion aftereffects. This example of a higher-level visual processing aftereffect without awareness may be attributed to the role of amygdala in non-conscious emotion processing. For example, Jiang and He (2006) demonstrated that fMRI BOLD amygdala responses to fearful faces were strong for both visible and invisible conditions using CFS, and reduced, though still evident, for neutral faces when awareness was suppressed. Fusiform face area was reduced, though still significant, for both face emotions when awareness was removed. Conversely, superior temporal sulcus responded only to fearful faces when awareness was suppressed. As such, the prominent view of aftereffects in interocular suppression studies appears to hold to the view that low-level visual attributes can escape suppression while higher-level visual stimuli that rely on processing further along the visual hierarchy have a stronger suppression effect (Nguyen et al., 2003; Yang et al., 2014).

### Conclusion

Understanding the underlying mechanism of aftereffects remains an elusive endeavor; one made more difficult by the likely different combination of higher- and lower-level cortical and subcortical processes involved depending on the complexity of the adapting stimulus (Webster, 2011). Nevertheless, the current study has shown that size aftereffects are not demonstrated once awareness has been suppressed by CFS. Based on our results, and other lines of evidence presented elsewhere, we argue that the gain mechanisms in V1 described by Pooresmaeili et al. (2013) are insufficient for driving size aftereffects and that they may also require reentrant or top–down influence from higher order visual cortical processing.

## Author Contributions

JS contributed to the experimental design, data collection, data analysis and the writing of the manuscript while RL, IS, and PC contributed to the experimental design, data analysis and the writing of the manuscript.

## Conflict of Interest Statement

The authors declare that the research was conducted in the absence of any commercial or financial relationships that could be construed as a potential conflict of interest.

## References

[B1] Adams W. J. Gray K. L. Garner M. Graf E. W. (2010). High-level face adaptation without awareness. *Psychol. Sci.* 21 205–210. 10.1177/0956797609359508 20424046

[B2] Addams R. (1834). An account of a peculiar optical phenomenon seen after having looked at a moving body, etc. *Lond. Edinb. Philos. Mag. J. Sci.* 5 373–374.

[B3] Almeida J. Mahon B. Z. Nakayama K. Caramazza A. (2008). Unconscious processing dissociates along categorical lines. *Proc. Natl. Acad. Sci. U.S.A.* 105 15214–15218. 10.1073/pnas.0805867105 18809923 PMC2567517

[B4] Almeida J. Pajtas P. E. Mahon B. Z. Nakayama K. Caramazza A. (2013). Affect of the unconscious: visually suppressed angry faces modulate our decisions. *Cogn. Affect. Behav. Neurosci.* 13 94–101. 10.3758/s13415-012-0133-7 23224765 PMC4752568

[B5] Amihai I. Deouell L. Bentin S. (2011). Conscious awareness is necessary for processing race and gender information from faces. *Conscious. Cogn.* 20 269–279. 10.1016/j.concog.2010.08.004 20843704 PMC3015017

[B6] Anderson E. J. Dakin S. C. Rees G. (2009). Monocular signals in human lateral geniculate nucleus reflect the Craik-Cornsweet-O’Brien effect. *J. Vis.* 9 14.11–14.18 10.1167/9.12.14 20053105

[B7] Badcock D. Clifford C. (2004). “The inputs to global form detection,” in *Seeing Spatial Form*, eds Jenkins M. Harris L. (Oxford: Oxford University Press), 37–50.

[B8] Bahmani H. Murayama Y. Logothetis N. K. Keliris G. A. (2014). Binocular flash suppression in the primary visual cortex of anesthetized and awake macaques. *PLOS ONE* 9:e107628 10.1371/journal.pone.0107628 PMC4162631 25216188

[B9] Chung C. Y. Khuu S. K. (2014). The processing of coherent global form and motion patterns without visual awareness. *Front. Psychol.* 5:195 10.3389/fpsyg.2014.00195 PMC3953956 24672494

[B10] Clifford C. W. Webster M. A. Stanley G. B. Stocker A. A. Kohn A. Sharpee T. O. (2007). Visual adaptation: neural, psychological and computational aspects. *Vision Res.* 47 3125–3131. 10.1016/j.visres.2007.08.023 17936871

[B11] Cohen J. (1988). *Statistical Power Analysis for the Behavioral Sciences.* Hillsdale, NJ: Lawrence Erlbaum Associates.

[B12] Cohen M. A. Nakayama K. Konkle T. Stantic M. Alvarez G. A. (2015). Visual awareness is limited by the representational architecture of the visual system. *J. Cogn. Neurosci.* 27 2240–2252. 10.1162/jocn_a_00855 26226078

[B13] Costa T. L. Costa M. F. Magalhaes A. Rego G. G. Nagy B. V. Boggio P. S. (2015). The role of early stages of cortical visual processing in size and distance judgment: a transcranial direct current stimulation study. *Neurosci. Lett.* 588 78–82. 10.1016/j.neulet.2014.12.055 25556682

[B14] Dehaene S. Naccache L. (2001). Towards a cognitive neuroscience of consciousness: basic evidence and a workspace framework. *Cognition* 79 1–37. 10.1016/S0010-0277(00)00123-2 11164022

[B15] Dehaene S. Sergent C. Changeux J. P. (2003). A neuronal network model linking subjective reports and objective physiological data during conscious perception. *Proc. Natl. Acad. Sci. U.S.A.* 100 8520–8525. 10.1073/pnas.1332574100 12829797 PMC166261

[B16] Eger E. Kell C. A. Kleinschmidt A. (2008). Graded size sensitivity of object-exemplar-evoked activity patterns within human LOC subregions. *J. Neurophysiol.* 100 2038–2047. 10.1152/jn.90305.2008 18632884

[B17] Ehrenstein W. H. Ehrenstein A. (1999). “Psychophysical methods,” in *Modern Techniques in Neuroscience Research*, eds Windhorst U. Johansson H. (Berlin: Springer), 1211–1241. 10.1007/978-3-642-58552-4_43

[B18] Faivre N. Berthet V. Kouider S. (2012). Nonconscious influences from emotional faces: a comparison of visual crowding, masking, and continuous flash suppression. *Front. Psychol.* 3:129 10.3389/fpsyg.2012.00129 PMC3342619 22563325

[B19] Fang F. Boyaci H. Kersten D. Murray S. O. (2008). Attention-dependent representation of a size illusion in human V1. *Curr. Biol.* 18 1707–1712. 10.1016/j.cub.2008.09.025 18993076 PMC2638992

[B20] Fogelson S. V. Kohler P. J. Miller K. J. Granger R. Tse P. U. (2014). Unconscious neural processing differs with method used to render stimuli invisible. *Front. Psychol.* 5:601 10.3389/fpsyg.2014.00601 PMC4058905 24982647

[B21] Gayet S. Van Der Stigchel S. Paffen C. L. (2014). Breaking continuous flash suppression: competing for consciousness on the pre-semantic battlefield. *Front. Psychol.* 5:460 10.3389/fpsyg.2014.00460 PMC4033185 24904476

[B22] Grill-Spector K. Kushnir T. Edelman S. Avidan G. Itzchak Y. Malach R. (1999). Differential processing of objects under various viewing conditions in the human lateral occipital complex. *Neuron* 24 187–203. 10.1016/S0896-6273(00)80832-6 10677037

[B23] Harris J. J. Schwarzkopf D. S. Song C. Bahrami B. Rees G. (2011). Contextual illusions reveal the limit of unconscious visual processing. *Psychol. Sci.* 22 399–405. 10.1177/0956797611399293 21317371 PMC3278746

[B24] Hesselmann G. Malach R. (2011). The link between fMRI-BOLD activation and perceptual awareness is “stream-invariant” in the human visual system. *Cereb. Cortex* 21 2829–2837. 10.1093/cercor/bhr085 21515713

[B25] Hubel D. H. Wiesel T. N. (1962). Receptive fields, binocular interaction and functional architecture in the cat’s visual cortex. *J. Physiol.* 160 106–154. 10.1113/jphysiol.1962.sp006837 14449617 PMC1359523

[B26] Jiang Y. He S. (2006). Cortical responses to invisible faces: dissociating subsystems for facial-information processing. *Curr. Biol.* 16 2023–2029. 10.1016/j.cub.2006.08.084 17055981

[B27] Kaunitz L. Fracasso A. Melcher D. (2011). Unseen complex motion is modulated by attention and generates a visible aftereffect. *J. Vis.* 11:10 10.1167/11.13.10 22072730

[B28] Lamme V. A. (2006). Towards a true neural stance on consciousness. *Trends Cogn. Sci.* 10 494–501. 10.1016/j.tics.2006.09.001 16997611

[B29] Lamme V. A. Roelfsema P. R. (2000). The distinct modes of vision offered by feedforward and recurrent processing. *Trends Neurosci.* 23 571–579. 10.1016/S0166-2236(00)01657-X 11074267

[B30] Laycock R. Chan D. Crewther S. G. (2017). Attention orienting in response to non-conscious hierarchical arrows: individuals with higher autistic traits differ in their global/local bias. *Front. Psychol.* 8:23 10.3389/fpsyg.2017.00023 PMC5241281 28149288

[B31] Lehmkuhle S. W. Fox R. (1975). Effect of binocular rivalry suppression on the motion aftereffect. *Vision Res.* 15 855–859. 10.1016/0042-6989(75)90266-7 1154668

[B32] Leopold D. A. (2012). Primary visual cortex: awareness and blindsight. *Annu. Rev. Neurosci.* 35 91–109. 10.1146/annurev-neuro-062111-150356 22715879 PMC3476047

[B33] Leopold D. A. Logothetis N. K. (1996). Activity changes in early visual cortex reflect monkeys’ percepts during binocular rivalry. *Nature* 379 549–553. 10.1038/379549a0 8596635

[B34] Ludwig K. Hesselmann G. (2015). Weighing the evidence for a dorsal processing bias under continuous flash suppression. *Conscious. Cogn.* 35 251–259. 10.1016/j.concog.2014.12.010 25649867

[B35] Ludwig K. Sterzer P. Kathmann N. Hesselmann G. (2016). Differential modulation of visual object processing in dorsal and ventral stream by stimulus visibility. *Cortex* 83 113–123. 10.1016/j.cortex.2016.07.002 27504609

[B36] Maier A. Wilke M. Aura C. Zhu C. Ye F. Q. Leopold D. A. (2008). Divergence of fMRI and neural signals in V1 during perceptual suppression in the awake monkey. *Nat. Neurosci.* 11 1193–1200. 10.1038/nn.2173 18711393 PMC2754054

[B37] Mancini F. Bolognini N. Bricolo E. Vallar G. (2011). Cross-modal processing in the occipito-temporal cortex: a TMS study of the Muller-Lyer illusion. *J. Cogn. Neurosci.* 23 1987–1997. 10.1162/jocn.2010.21561 20807050

[B38] Maruya K. Watanabe H. Watanabe M. (2008). Adaptation to invisible motion results in low-level but not high-level aftereffects. *J. Vis.* 8 7 1–11. 10.1167/8.11.7 18831601

[B39] Masuda A. Watanabe J. Terao M. Watanabe M. Yagi A. Maruya K. (2011). Awareness of central luminance edge is crucial for the Craik-O’Brien-Cornsweet effect. *Front. Hum. Neurosci.* 5:125 10.3389/fnhum.2011.00125 PMC3203414 22059072

[B40] Miles W. R. (1930). Ocular dominance in human adults. *J. Gen. Psychol.* 3 412–430. 10.1080/00221309.1930.9918218

[B41] Moradi F. Koch C. Shimojo S. (2005). Face adaptation depends on seeing the face. *Neuron* 45 169–175. 10.1016/j.neuron.2004.12.018 15629711

[B42] Nguyen V. A. Freeman A. W. Alais D. (2003). Increasing depth of binocular rivalry suppression along two visual pathways. *Vision Res.* 43 2003–2008. 10.1016/S0042-6989(03)00314-6 12842153

[B43] Noguchi Y. Yokoyama T. Suzuki M. Kita S. Kakigi R. (2012). Temporal dynamics of neural activity at the moment of emergence of conscious percept. *J. Cogn. Neurosci.* 24 1983–1997. 10.1162/jocn_a_00262 22721378

[B44] Oldfield R. C. (1971). The assessment and analysis of handedness: the Edinburgh inventory. *Neuropsychologia* 9 97–113. 10.1016/0028-3932(71)90067-4 5146491

[B45] O’Shea R. P. Crassini B. (1981). Interocular transfer of the motion after-effect is not reduced by binocular rivalry. *Vision Res.* 21 801–804. 10.1016/0042-6989(81)90177-2 7314456

[B46] Pascual-Leone A. Walsh V. (2001). Fast backprojections from the motion to the primary visual area necessary for visual awareness. *Science* 292 510–512. 10.1126/science.1057099 11313497

[B47] Pooresmaeili A. Arrighi R. Biagi L. Morrone M. C. (2013). Blood oxygen level-dependent activation of the primary visual cortex predicts size adaptation illusion. *J. Neurosci.* 33 15999–16008. 10.1523/JNEUROSCI.1770-13.2013 24089504 PMC4888977

[B48] Purves D. Shimpi A. Lotto R. B. (1999). An empirical explanation of the cornsweet effect. *J. Neurosci.* 19 8542–8551. 10493754 10.1523/JNEUROSCI.19-19-08542.1999 PMC6783017

[B49] Stein T. Sterzer P. (2011). High-level face shape adaptation depends on visual awareness: evidence from continuous flash suppression. *J. Vis.* 11:5 10.1167/11.8.5 21742962

[B50] Stein T. Sterzer P. (2014). Unconscious processing under interocular suppression: getting the right measure. *Front. Psychol.* 5:387 10.3389/fpsyg.2014.00387 PMC4018522 24834061

[B51] Sterzer P. Jalkanen L. Rees G. (2009). Electromagnetic responses to invisible face stimuli during binocular suppression. *Neuroimage* 46 803–808. 10.1016/j.neuroimage.2009.02.046 19285140

[B52] Sterzer P. Stein T. Ludwig K. Rothkirch M. Hesselmann G. (2014). Neural processing of visual information under interocular suppression: a critical review. *Front. Psychol.* 5:453 10.3389/fpsyg.2014.00453 PMC4032950 24904469

[B53] Suzuki S. Cavanagh P. (1998). A shape-contrast effect for briefly presented stimuli. *J. Exp. Psychol. Hum. Percept. Perform.* 24 1315–1341. 10.1037/0096-1523.24.5.1315 9778826

[B54] Sweeny T. D. Grabowecky M. Suzuki S. (2011). Awareness becomes necessary between adaptive pattern coding of open and closed curvatures. *Psychol. Sci.* 22 943–950. 10.1177/0956797611413292 21690314 PMC3261759

[B55] Thompson P. Burr D. (2009). Visual aftereffects. *Curr. Biol.* 19 R11–R14. 10.1016/j.cub.2008.10.014 19138580

[B56] Tong F. (2003). Primary visual cortex and visual awareness. *Nat. Rev. Neurosci.* 4 219–229. 10.1038/nrn1055 12612634

[B57] Tootell R. B. Hamilton S. L. Switkes E. (1988). Functional anatomy of macaque striate cortex. IV. Contrast and magno-parvo streams. *J. Neurosci.* 8 1594–1609. 3367212 10.1523/JNEUROSCI.08-05-01594.1988 PMC6569196

[B58] Troiani V. Schultz R. T. (2013). Amygdala, pulvinar, and inferior parietal cortex contribute to early processing of faces without awareness. *Front. Hum. Neurosci.* 7:241 10.3389/fnhum.2013.00241 PMC3674317 23761748

[B59] Tsuchiya N. Koch C. (2005). Continuous flash suppression reduces negative afterimages. *Nat. Neurosci.* 8 1096–1101. 10.1038/nn1500 15995700

[B60] Van Essen D. C. Gallant J. L. (1994). Neural mechanisms of form and motion processing in the primate visual system. *Neuron* 13 1–10. 10.1016/0896-6273(94)90455-3 8043270

[B61] Virsu V. (1978). Retinal mechanisms of visual adaptation and afterimages. *Med. Biol.* 56 84–96. 661403

[B62] Wade N. J. Wenderoth P. (1978). The influence of colour and contour rivalry on the magnitude of the tilt after-effect. *Vision Res.* 18 827–835. 10.1016/0042-6989(78)90123-2 676090

[B63] Wang L. Weng X. He S. (2012). Perceptual grouping without awareness: superiority of Kanizsa triangle in breaking interocular suppression. *PLOS ONE* 7:e40106 10.1371/journal.pone.0040106 PMC3387015 22768232

[B64] Watanabe M. Cheng K. Murayama Y. Ueno K. Asamizuya T. Tanaka K. (2011). Attention but not awareness modulates the BOLD signal in the human V1 during binocular suppression. *Science* 334 829–831. 10.1126/science.1203161 22076381

[B65] Webster M. A. (2011). Adaptation and visual coding. *J. Vis.* 11:3 10.1167/11.5.3 PMC3245980 21602298

[B66] Wetzels R. Matzke D. Lee M. D. Rouder J. N. Iverson G. J. Wagenmakers E.-J. (2011). Statistical evidence in experimental psychology: an empirical comparison using 855 t tests. *Perspect. Psychol. Sci.* 6 291–298. 10.1177/1745691611406923 26168519

[B67] Wilke M. Logothetis N. K. Leopold D. A. (2006). Local field potential reflects perceptual suppression in monkey visual cortex. *Proc. Natl. Acad. Sci. U.S.A.* 103 17507–17512. 10.1073/pnas.0604673103 17088545 PMC1859959

[B68] Wood D. K. Chouinard P. A. Major A. J. Goodale M. A. (2016). Sensitivity to biomechanical limitations during postural decision-making depends on the integrity of posterior superior parietal cortex. *Cortex* 10.1016/j.cortex.2016.07.005 [Epub ahead of print]. 27477623

[B69] Yang E. Brascamp J. Kang M.-S. Blake R. (2014). On the use of continuous flash suppression for the study of visual processing outside of awareness. *Front. Psychol.* 5:724 10.3389/fpsyg.2014.00724 PMC4093749 25071685

[B70] Yuval-Greenberg S. Heeger D. J. (2013). Continuous flash suppression modulates cortical activity in early visual cortex. *J. Neurosci.* 33 9635–9643. 10.1523/JNEUROSCI.4612-12.2013 23739960 PMC3760788

[B71] Zhao C. Series P. Hancock P. J. Bednar J. A. (2011). Similar neural adaptation mechanisms underlying face gender and tilt aftereffects. *Vision Res.* 51 2021–2030. 10.1016/j.visres.2011.07.014 21810438

